# Twelve Years Since Importance of Cross-Cultural Competency Recognized: Where Are We Now?

**DOI:** 10.5811/westjem.2016.10.31780

**Published:** 2016-12-06

**Authors:** Remi A. Kessler, Wendy C. Coates, Arjun Chanmugam

**Affiliations:** * Johns Hopkins School of Medicine, Department of Emergency Medicine, Baltimore, Maryland; † University of California, Los Angeles, David Geffen School of Medicine at UCLA, Harbor-UCLA Department of Emergency Medicine, Los Angeles, California

## Abstract

**Introduction:**

The objective of this study was to analyze the content and volume of literature that has been written on cultural competency in emergency medicine (EM) since its educational imperative was first described by the Institute of Medicine in 2002.

**Methods:**

We conducted a comprehensive literature search through the PubMed portal in January 2015 to identify all articles and reviews that addressed cultural competency in EM. Articles were included in the review if cultural competency was described or if its impact on healthcare disparities or curriculum development was described. Two reviewers independently investigated all relevant articles. These articles were then summarized.

**Results:**

Of the 73 abstracts identified in the initial search, only 10 met criteria for inclusion. A common theme found among these 10 articles is that cultural competency in EM is essential to reducing healthcare disparities and improving patient care. These articles were consistent in their support for cross-cultural educational advancements in the EM curriculum.

**Conclusion:**

Despite the documented importance of cultural competency education in medicine, there appears to be only 10 articles over the past 12 years regarding its development and implementation in EM. This comprehensive literature review underscores the relative dearth of publications related to cultural competency in EM. The limited number of articles found is striking when compared to the growth of EM research over the same time period and can serve as a stimulus for further research in this significant area of EM education.

## INTRODUCTION

Emergency departments (ED) are experiencing an increasingly diverse patient population, both racially and culturally.^1^,^2^ In 2002, the now-famous Institute of Medicine (IOM) report entitled “Unequal Treatment: Confronting Racial and Ethnic Disparities in Health Care” addressed this issue and emphasized the need to improve healthcare disparities.^3^ By 2003, the emergency medicine (EM) literature started to address the issue of disparities in EM care and the need for workforce diversity and training.^4^ In the same year, cross-cultural competency recommendations were made in terms of EM educational curricula.^5^ Although the initial response to disparities and cross-cultural competency training was noteworthy, it is unclear how much this issue has been advanced by EM educators and researchers since the first papers were published in this area.

The ED is frequently the first point of access to care for many minority groups.^6^ Awareness of cultural sensitivities, or cultural competency, is necessary to overcome bias and clinical uncertainty that is often experienced by those treating these patients.^1^,^2^ An important point is that different cultures embody divergent help-seeking behaviors. This concept is well summarized from the textbook of *Emergency Psychiatry* in which Dr. Jayaram writes, “Notions of sickness are derived from systems of medical understanding that exist within a culture. Beyond that both the provider and the patient have epistemic systems that dictate how individuals express suffering.”^7^ It takes effort to bridge the differences in attitudes of illness between provider and patient, which are exacerbated when the two are from distinct and unfamiliar cultural backgrounds.

Effective communication between patient and physician is commonly regarded as a primary method to overcome cultural difference. Communication is dependent on mutually understood social constructs; however, these social constructs become ambiguous when the provider is unaware, or otherwise fails to recognize, that these cultural differences exist. Provider values, as well as patient values, can influence interpretation of symptoms and patient compliance with medical interventions.^8^ These values are expressed both verbally and nonverbally. Miscommunication is exacerbated when there is language discordance as well underappreciated cultural manifestations of illness and health.^9^

Cultural differences can serve as a prelude to biases, which can be defined as “prejudiced or partial viewpoints that affects someone’s interpretation of a problem.”^10^ A lack of appreciation for a specific culture can result in assumptions and subsequent management errors on the part of the culturally unacquainted provider. These cognitive biases serve as impediments to communication and as a result impair the achievement of an accurate diagnostic hypothesis. Cultural competency helps physicians to overcome these biases.^1^ Thus, improving cultural competency in EM faculty and residents can help to ameliorate biases, which in turn may improve patient outcomes and the patient experience.

EM has exploded with available information and knowledge in many different domains since the inception of the specialty in 1961. With the growth in literature in EM topics, has the research regarding cultural competency education in EM, and its correlation with reducing bias and improving patient outcomes in the ED grown accordingly? This comprehensive literature review seeks to assess the literature and provide a brief summary of the findings associated with cross-cultural competency in EM since the IOM report first described this educational imperative.

## METHODS

We conducted a comprehensive literature search to identify articles and reviews that address cultural competency in EM. This included articles that were focused on any cultural competency education measures for EM faculty and residents. We also included any article in which the impact on health disparities and/or establishing an EM curriculum was discussed. We performed an electronic search through PubMed in January 2015 and selected the terms “emergency medicine” or other common words used to describe an ED, coupled with “cultural competency” or “cross-cultural training” or “cross-cultural communication” or “cultural disparities,” or other comparable variations to expand the search. We limited our search to English-language reviews and journal articles only. We evaluated all applicable papers for their relevance to EM cross-cultural training and associated curriculum development. In addition, the references from these papers were examined for other potential sources of information. The chosen articles were carefully scrutinized and their information was extracted to provide a brief comprehensive summary of cross-cultural competency in EM since its significance was first identified. The initial search revealed 73 articles. Two reviewers independently examined the search results to screen for applicable articles. Articles were targeted for inclusion only if they met the following criteria:

1. U.S. or Canadian-based studies;
2. Adult emergency medicine focused;
3. Some link to cultural competency, cultural awareness, diversity, cultural sensitivity, or multicultural education;
4. Medical journal (non-nursing or allied health).

## RESULTS

There was agreement on 10 articles (Table), with three articles in question. After abstract review, we excluded the three articles because they documented a need for cultural sensitivity and training but not how it should be addressed in the EM curriculum. Of the excluded papers, Aratani and Addy concluded that **“**the disparities indicate a need for culturally sensitive and gender-specific services for this vulnerable population [at-risk youth].”^11^ Royl et al. proposed that culturally sensitive healthcare might be enhanced through the use of interpreters, standardized surveys, and ease of access to appropriate cranial imaging in cases in which a benign etiology in headache cannot be confidently concluded. 12 The final article in question, Greenberg and Pierog, indicated that ACLS provider and instructor materials do not depict a fair representation of minority populations.^13^

The 10 articles that met criteria and selected by both reviewers are listed in the table.

## DISCUSSION

Different cultures view illness and its effects in their own distinct manner. Although suffering can be considered as a universally recognizable situation, the type of suffering and the extent of suffering due to illness is variable in different cultures. Language discordance and a lack of appreciation for the variation of cultural manifestation of illness can predispose one’s thinking in favor of a certain viewpoint over more appropriate viewpoints. Helping providers to overcome cultural biases has been recognized as an important education goal in EM.^14^

It is well understood that preconceived notions about the behavior or health of minority populations and prejudice are contributing factors to the disparities observed in healthcare.^3^ Intrinsic bias on the part of the provider, or cognitive dispositions to respond (CDR), may contribute to flawed clinical reasoning and diagnostic errors.^15^ These are likely to be exacerbated when confronted with cultural differences unfamiliar to the provider. The tendency to adopt a predetermined viewpoint about a patient based on sociocultural factors, whether conscious or unconscious, interferes with a physician’s ability to create an appropriate therapeutic plan. In addition, the time pressures and demanding nature of the ED atmosphere do not always allow for mindfulness, or presence in the moment. This presence in the moment is an important prerequisite in gaining a better understanding of a patient’s background and the patient’s behavior toward illness. A clear understanding of the patient’s behavior helps to avoid stereotyping, which is often at its peak during multi-tasking, stressful events, and under time-sensitive situations.^4^ As such, the importance of cross-cultural competency is amplified in specialties such as EM that are constrained by time sensitivities. The manner in which it is taught and when it is taught in the EM curriculum needs to be clarified.

Among the 10 articles that met criteria for inclusion, four were particularly pertinent in summarizing the imminent necessity of developing cultural competency measures and offered multiple concrete solutions to address this need in EM education. In 2003, Cone et al. and Hamilton and Marco first introduced the importance of cross-cultural training in EM as a mechanism of reducing disparity and its ties with educational initiatives.^4^,^5^ These articles emphasized the extent of the issue of healthcare disparities as outlined in the IOM report. They suggested an increase in workforce diversity and a cultural competency curriculum in EM as potential resolutions. In 2003, the Society of Academic Emergency Medicine (SAEM) and the Council of Emergency Medicine Residency Directors (CORD) established the Cultural Competency Curriculum Task Force (CCCTF) with the objective of developing a model curriculum for residency programs.^4^ One of the papers referred to a web-based resource at the University of Virginia School of Medicine website. Within the website (med-ed.virgina.edu) is a page that is self-described as a monograph on cultural competency and is attributed to the CORD/SAEM Diversity Task force, also known as the CCCTF.^16^ The website currently offers instructional materials, including 13 example clinical cases, and has chapters describing cultural competency as it pertains to EM. It also has links to numerous other relevant publications available in online format.

In 2006, Hobgood et al. provided a detailed review of the educational models in practice in all fields of medicine for teaching cultural competency as well as the barriers that impede the establishment of cross-cultural education.^17^ Their paper described curricular methods for cross-cultural training employed in medical schools and residencies that include cultural immersion, community clinical experience, simulation, didactic models, literary models, portfolios, and continuing medical education adjuncts. They presented a cultural competency measure uniquely intended to educate faculty members in workshop format. The authors also remarked that this type of periodic and recurrent model would complement EM conference scheduling if it were to be extrapolated for EM education. In addition, the paper recognized a mixed-method instructional program that assesses students by measurable competencies. This paper also identified immersion models, whereby a group of students spends either a short-term or extensive period of time in a foreign location to foster cultural awareness and understanding.

Additionally, it stated that the Association of American Medical Colleges offers short strategies to assist in cultural information gathering during an initial physician-patient encounter. The paper also identified existing methods used for assessment including the Betancourt model and the Accreditation Council of Graduate Medical Education (ACGME) Toolbox. They described the Betancourt model as a system that evaluates attitude, knowledge, and skill using several ways to score each category, while the ACGME Toolbox provides a plethora of alternative methods. Supporting the Hobgood et al. paper was a statement in 2008 by the American College of Emergency Physicians (ACEP) affirming that “cultural awareness should be an essential element in the training of healthcare professionals and to the provision of safe quality care in the ED environment.”^18^

Padela and Punekar in 2008 emphasized the significance of cultural sensitivity in the ED environment, and presented three ways to improve minority outcomes through teachings of cross-cultural communication:^1^ 1) increasing cross-cultural training and decreasing physician bias; 2) maximizing provider diversity; and 3) accommodating diverse patients’ needs. Bowman et al. in 2011 discussed the possibility of initiating an EM cultural competency curriculum and the obstacles associated with its implementation.^14^ These results were obtained from a CORD workshop survey, and the authors used an implicit association test (IAT) to investigate bias in its survey participants. They chose to administer this tool due to the realization of the growing importance of addressing unconscious bias in cultural competency acquisition. Their primary notion was that bias is present and active in even the most well-intentioned physician and overcoming that bias can be challenging to effectively address in any cross-cultural training curriculum. In their paper, workshop participants came to a consensus that overcoming personal biases was a necessity in order to ameliorate cultural competency education. Participants also described obstacles that might be experienced in attempting to inaugurate such interventions. These barriers increased in complexity at institutions in which faculty, residents, and patients are less diverse and in which minority faculty do not wish to possess the burden of acting as the sole resource in cross-cultural education. In the paper, the participants reached agreement in that minority faculty should not solely be held accountable for amending the curriculum. Participants also expressed interest in developing cultural competency curricula by non-program director faculty members, and some intended to start discussions with program leadership. In contrast, some attendees felt troubled in asking for curricular modifications at institutions in which there was a scarcity of resources for this type of programming. Additionally, some noted that negative behaviors toward certain groups of patients were tolerated at some institutions.

Finally, in 2005 it was found that resident physicians in EM were more likely to disclose a deficiency in cultural competency education when compared with residents from other clinical areas.^19^ This paper was not included in the 10-paper summary table as it was not EM focused and did not meet selection criteria.

Fortunately, the EM Milestones do address cultural competency in at least two domains. In EM Milestone 20, Professional Values (PROF1) Level 1, there is a statement indicating that behavior that “conveys caring, honesty, genuine interest and tolerance when interacting with a diverse population of patients and families” must be shown. Additionally, EM Milestone 22 – Patient-Centered Communication (ICS1) Level 3 – requires that residents be able to “effectively communicate with vulnerable populations, including both patients at risk and their families.”

## LIMITATIONS

There are several limitations in this study. First, due to the specifications employed in the title and abstract review process, it is possible that relevant journal articles that did not meet our defined criteria were excluded. By ensuring that the criteria were broad enough to encompass all aspects of the topic discussed in this paper, we attempted to minimize the possible effects of this. Second, only two reviewers were responsible for screening articles, which may have resulted in selection bias or bias due to too few reviewers. In an attempt to diminish the selection bias, the two reviewers conducted independent screenings of the articles, then compared and discussed findings. Additionally, our search was PubMed-based and did not include articles from other databases. A preliminary review of EMBASE and PSYCINFO revealed no relevant articles but did reveal two brief published abstracts.

A critical limitation in this study was that advances in the area of cross-cultural competency may have been made by individual training programs or other forums in which we were not able to assess or include in this literature review. It may be that programs are adequately addressing the educational imperative of cross-cultural competency, but their methods and findings are not well published or were not discovered using the search strategy we employed.

## CONCLUSION

Cultural competency has been recognized as an important educational goal for physicians and physician training since the IOM report in 2002. During the 12 years since that report, 10 papers have been published describing the significance and value of cultural competency in EM education. Given the importance of this topic, as evidenced by the IOM report and the subsequent papers on the topic, the volume of literature describing educational advances in this area appears to be relatively light. Our hope is that this comprehensive review will spur publications and additional attention to the area of cultural competency in EM. As has been stated in the literature, cross-cultural competency is an important means of improving patient safety and is a critical tool in creating a more effective and therapeutic patient experience in the emergency medicine setting.

## Figures and Tables

**Table:** Articles addressing cross-cultural competency in emergency medicine.

Date	Author	Title	Main points
November 2003	Cone DC, Richardson LD, Todd KH, Betancourt JR, Lowe RA.	Healthcare Disparities in Emergency Medicine	Addresses issues of disparities in healthcare and makes recommendations.
November 2003	Hamilton G, Marco C.	Emergency Medicine Education and Health Care Disparities	Provides rationale for cultural diversity in EM training and suggests educational approaches.
August 2006	Sheridan I.	Treating the World Without Leaving Your ED: Opportunities to Deliver Culturally Competent Care	Explains challenges faced by immigrant groups and their physicians in clinical encounters.
December 2006	Hobgood C, Sawning S, Bowen J, Savage K.	Teaching Culturally Appropriate Care: A Review of Educational Models and Methods	Presents overview of educational models for cultural training and EM applicability.
March 2007	Padela Al.	Can You Take Care of My Mother? Reflections on Cultural Competency and Clinical Accommodation	Portrays account of cross-cultural care.
August 2008	ACEP	Cultural Awareness and Emergency Care	Concludes that cultural sensitivity is necessary in EM training and in the practice of EM.
January 2009	Padela Al, Punekar IR.	Emergency Medical Practice: Advancing Cultural Competence and Reducing Health Care Disparities	Highlights importance of cultural awareness in the ED and addresses bias, cultural training, and workforce diversity.
October 2011	Bowman SH, Moreno-Walton L, Ezenkwele UA, Heron SL.	Diversity in Emergency Medicine Education: Expanding the Horizon	Discusses results of a survey testing unconscious bias and possibility of initiating an EM curriculum on cultural competency.
August 2013	Ezenkwele UA, Roodsari, GS.	Cultural Competencies in Emergency Medicine: Caring for Muslim-American Patients from the Middle East	Presents guideline to overcoming cultural barriers to effectively treat this population.
May 2014	Moll J, Kreiger P, Moreno-Walton L, Lee B, Slaven E, James T, Hills D, Podolsky S, Corbin T, Heron SL.	The Prevalence of Lesbian, Gay, Bisexual, and Transgender (LBGT) Health Education and Training in Emergency Medicine Residency Programs: What Do We Know?	Describes survey about LGBT education and found that many EM programs do not have a curriculum on this issue.

*EM,* emergency medicine; *ED,* emergency department
